# Human Epidermal Growth Factor Receptor 2 Status in Gastric Carcinomas with Distinctive Prevalent Cribriform Component

**DOI:** 10.1155/2018/1505428

**Published:** 2018-02-18

**Authors:** Antonio Ieni, Giuseppe Angelico, Valeria Barresi, Giuseppe Giuffrè, Francesco Arena, Rosario Alberto Caruso, Giovanni Tuccari

**Affiliations:** Section of Anatomic Pathology, Azienda Ospedaliera Universitaria “Gaetano Martino” and Department of Human Pathology “Gaetano Barresi”, University of Messina, 98123 Messina, Italy

## Abstract

**Objectives:**

A cribriform architectural pattern has been reported in 9% of one unselected consecutively collected series of gastric carcinomas (GC) with unfavourable prognostic outcome. Taking into consideration the biological relevance of the human epidermal growth factor receptor 2 (HER2) status, we have analyzed a cohort of GC with a cribriform component more than 40% (CGC) to evaluate the HER2 amplification rate as a potential target for therapy with trastuzumab.

**Results:**

HER2 overexpression was encountered in 21 of 100 (21%) GC; a progressive increase in HER2 amplification was appreciated moving from non-CGC (20.6%) towards CGC cases (21.6%), although this difference does not reach a statistical significance. Nevertheless, either in univariate or in multivariate analyses, stage and HER2 status showed a significant *p* value (<0.001) in CGC patients.

**Conclusions:**

Our data confirmed a worse prognosis in all CGC patients with HER2 amplification, resulting in a shorter survival time. We invite all pathologists in their daily practice to specify the occurrence of cribriform neoplastic component in GC, either in surgical or in bioptical samples, taking into practical assessment the high HER2 overexpression rate in order to correctly treat these patients with worse behavior.

## 1. Introduction

The cribriform histological pattern has been attributed to tumors showing an architecture made of straight packed glands with not uniform distributed lumina, without interposed stromal tissue [1, 2]. This peculiar pattern has been identified in invasive carcinomas rising in many different organs, such as prostate, breast, lung, colon, thyroid, skin, and stomach [2–7]. In this latter localization, a specific cribriform gastric carcinoma (CGC) has not been described in the last WHO classification of gastrointestinal tract tumors [8], even if the identification of cribriform pattern may have interesting practical prognostic implications for oncologists [1, 2]. In fact, it has been reported that CGC was associated with higher stage, lymphovascular and perineural invasion as well as with lower disease-free survival rate in comparison to conventional histotypes of gastric carcinomas (GC) [3]. In detail, the cribriform pattern has been reported in 9% of unselected consecutively collected casuistry of gastric carcinomas (GC) with unfavourable prognostic outcome [3]. By immunohistochemistry, neoplastic elements present in CGC were diffusely stained with CK7 and CK19, but focally for CK20 [3]. Moreover, MUC5AC has been also reported as positive, while hormone receptors, CDX2, MUC1, MUC2, and GCDFP-15, were always unexpressed [3].

Human epidermal growth factor receptor 2 (HER2) is a 185 kDa transmembrane tyrosine kinase receptor, member of the EGFR family, which plays a central role in growth factor signal transduction [9]. HER2 overexpression/amplification is involved in the development of various solid tumors, playing a pivotal role in oncogenic tumorogenesis and representing one of the most important therapeutic target in oncology [10].

A phase III randomized study (ToGA) demonstrated a significant survival benefit in patients affected by advanced GC with HER2 overexpression and treated with combined trastuzumab and chemotherapy, increasing the survival in GC [9, 10]. In detail, it has been shown that the combination of chemotherapy plus trastuzumab was statistically more advantageous than chemotherapy alone, with an increased median overall survival of nearly 3 months (OS 13.8 versus 11.1 months without trastuzumab), prolonging also the progression-free survival and the response rate in GC patients [9].

The rate of HER2 overexpression varies according to the histotype of GC, with higher frequency evidenced in the intestinal histotype (81.6%–91%) compared to the diffuse or mixed (4%–7.9%). Of note, the pattern of HER2 immunoreactivity is frequently heterogeneous in intestinal GC, which showed intermingled HER2-positive and HER2-negative areas [11]. On the other hand, a more uniform unreactive HER2 pattern was encountered in diffuse histotype [12, 13]. Although many authors have not clarified the potential prognostic HER2 value, a larger number of studies indicate that HER2 represents a negative prognostic factor, showing more aggressive biological behavior and higher frequency of recurrence in HER2-positive tumors [14–16]. In particular, it has been reported that HER2 overexpression rate progressively increases moving from the poorly cohesive WHO histotype to the mitochondrion-rich adenocarcinoma (MRC), tubular adenocarcinoma, and hepatoid carcinoma (HAS), which showed the highest frequency of HER2 positivity and the worst prognosis [12].

The aim of the present study is to firstly analyze the HER2 status in a cohort of selected CGC in order to evaluate the possible relationship with clinic-pathological characteristics as well as prognostic parameters such as disease-free interval and final outcome.

## 2. Materials and Methods

### 2.1. Patient Selection and Clinicopathologic Features

In the period 2006–2015, one hundred ten surgically treated GC with no neoadjuvant chemotherapy were selected from files of our department of the University of Messina, Azienda Ospedaliera Universitaria “Polyclinic G Martino” (Messina, Italy). The enrollment was designed in order to have a larger majority of intestinal type GC, in which the rate of CGC is more frequent; in addition, the accessibility of follow-up data has been considered a further element of choice.

The tumors were taken from an equal number of patients (58 men and 52 women), mean age 68 years (range 40–84 years). A Gaussian distribution was used to calculate the appropriate number of patients. 10 patients who died within 30 days after surgery (postoperative mortality) were excluded from the study. Tumor localization in the stomach was lower third in 57, middle third in 35, and upper third in 8 cases, five of which were located at the gastrooesophageal junction. For all cases, follow-up data were available, with a mean value 39.23 months (range 4–96 months), while the mean of CGC patients was 34.24 months. The patients' personal details were nonidentifiable, and all the patients had provided written consent to their medical information being used for research purposes, according with the Helsinki declaration.

All gastric surgical specimens had been 10% neutral formalin fixed for 24–48 hours and paraffin embedded at 56°C. Histotypes according to the WHO classification revealed 49 tubular/papillary/mucinous adenocarcinomas, 24 poorly cohesive carcinomas, and 37 cases of CGC variant. CGC variant was defined when at least 40% of the tumor exhibited the occurrence of identifiable neoplastic glands forming solid nests with round spaces (punch-out) leading to a cribriform pattern, similarly to that elsewhere reported [3]. This histological model greatly mimics the cribriform pattern occurring in breasts or salivary glands. The patients were staged using the seventh edition of the American Joint Committee on Cancer Tumor Node Metastasis (TNM) staging system [12]. Clinicopathological data of 100 GC patients were summarized in Table 1.

### 2.2. Immunohistochemical Procedures and Interpretation

HER2 status has been evaluated by immunohistochemical procedure on silane-coated slides carrying 3 mm thick sections using HercepTest (DAKO). An antigen retrieval pretreatment was performed by three changes in 0.01 M citrate buffer pH 6.0 in a microwave oven at 750 W. Each immunostained section was evaluated by the following score: 0 (absent staining); 1+ (faint and discontinuous membranous staining in 10% of neoplastic elements); 2+ (light to moderate lateral, basolateral, or complete membranous staining in 10% of neoplastic elements); and 3+ (strong, intense lateral, basolateral, or complete staining in 10% of neoplastic elements). All cases considered equivocal (2+) have been furtherly assessed by FISH test (pharmDx DAKO); nevertheless, as the presence of some IHC1+/FISH amplified cases has been reported elsewhere [10], 1+ cases were also submitted to the FISH procedure. Gene amplification was recorded when the HER2 to CEP17 signal ratio was 2.0 or greater.

Statistical analysis was performed by chi-square test to analyze associations between HER2 status and clinicopathological parameters. Cancer-specific survival analysis was performed by the Kaplan-Meier method, and for comparison of the survival curves, the Mantel-Cox log-rank test was used. A multivariate analysis (Cox regression model) was utilized to determine the independent effects of variables on overall survival. A *p* value less than 0.05 was considered statistically significant. Data were analyzed using the SPSS package V.6.1.3.

## 3. Results

Clinicopathological parameters as well as immunohistochemical data in relation to HER2 status are summarized in Table 1.

HER2 overexpression was encountered in 21 of 100 (21%) GC; in detail, 16 cases (16%) exhibited a 3+ score and 5 of 12 cases with a 2+ score showed HER2 amplification by the FISH test. Moreover, 66 cases exhibited 0 as the HER2 score (66%), while six carcinomas (6%) were scored as 1+, but none of these was amplified after the FISH test. A progressive increase in HER2 overexpression was appreciated moving from non-CGC (20.6%) towards CGC cases (21.6) (Table 1).

Regarding tumor stage in relation to HER2 status, GC were subdivided 69 in stage II (13% HER+), 28 stage III (39.3% HER2+), and 3 stage IV (33.3% HER2+); the *p* value was 0.014. Taking the clinical course into consideration, 29.5% of 61 patients died for GC showed HER2 expression/amplification; only 7.7% showed HER2 overexpression in 39 alive patients; this difference was statistically significant (*p* = 0.009) (Table 1).

**Figure 1:**
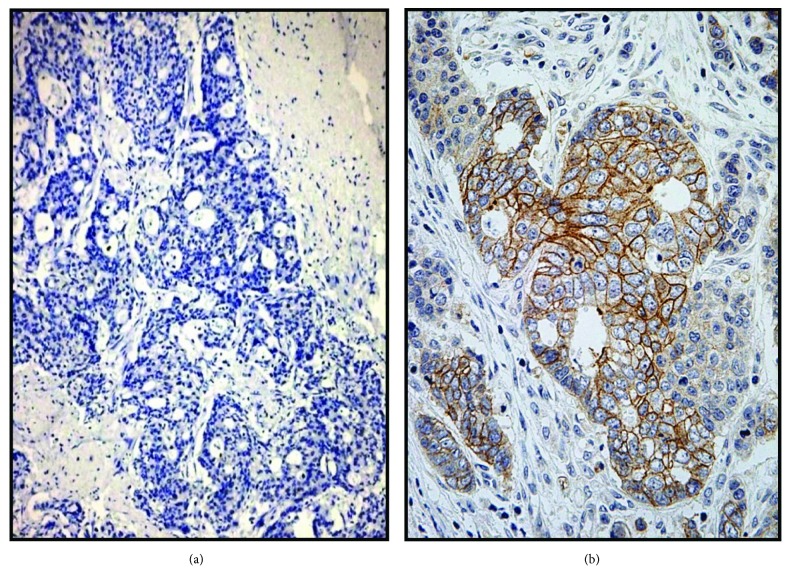
Cribriform gastric carcinoma (CGC): absence of HER2 immunoreactivity (a, original magnification ×160); a strong 3+ HER2 expression in another case (b, original magnification ×240) (Meier Haemalum nuclear counterstain).

When the analysis was limited to the CGC variant, the overall HER2 amplification concerns 8 out 37 cases (21.6%); in detail, 6/37 (16.22%) were scored as 3+ (Figure 1(a)), and 4/37 appeared equivocal with 2+ score, two of which showed FISH amplification. Finally, 3/37 (8.10%) were 1+ and 24/37 (64.86%) were categorized as 0 (Figure 1(b)).

**Figure 2:**
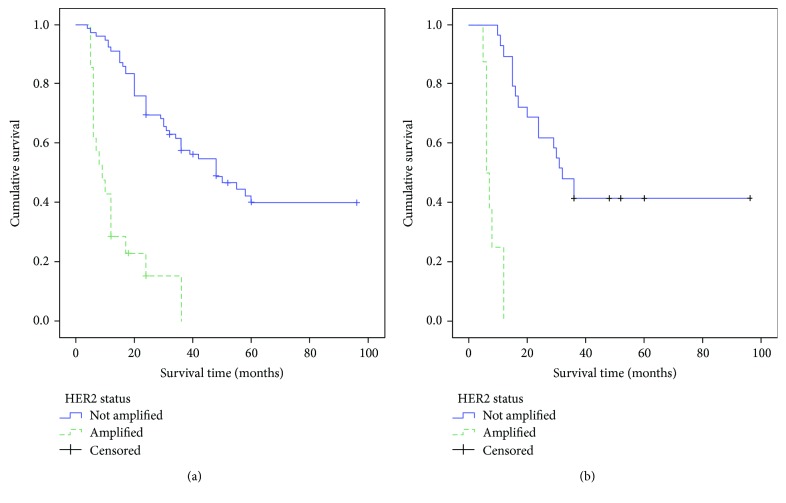
Overall survival curves of all cases of gastric adenocarcinomas (a) and of the cribriform (CGC) variant (b) according to HER2 status.

The survival curves of all patients as well as those of CGC patients, with or without HER2 overexpression, performed by the Kaplan-Meier method, were illustrated in (Figure 2).

In univariate analysis of all patients, stage (*χ*^2^ = 41.721) and HER2 status (*χ*^2^ = 45.754) showed a significant *p* value (<0.001); these two parameters maintained the same statistical significance when only CGC patients were considered (*χ*^2^ = 14.182) and (*χ*^2^ = 39.973), respectively (Table 2). By multivariate survival analysis, the independent prognostic value of HER2 amplification was confirmed together with the stage of GC (Table 3); moreover, similar results were obtained taking into consideration only patients affected by the CGC variant (Table 3).

## 4. Discussion

It is well known that HER2 overexpression in advanced gastric carcinomas has been considered an independent prognostic parameter, although its association with patient survival or tumor metastatic status is still controversial [10–13]. Moreover, a relationship between HER2 amplified status and high grade, stage, Ki67 value and death for GC has been previously elsewhere underlined [10–13]. In addition, it is noteworthy that the rate of HER2 overexpression varies according to the GC histotype, with higher frequency evidenced in intestinal histotype compared to the diffuse or mixed one [17–20]. Interestingly, in a previous analysis concerning unusual histotype of GC, we have already documented a progressive increase of HER2 amplification moving from the poorly cohesive histotype to the mitochondrion-rich adenocarcinoma, tubular adenocarcinoma, and hepatoid carcinoma, which showed the highest frequency of HER2 positivity and the worse prognosis [12, 21]. Until now, no data about HER2 status have been reported in gastric adenocarcinomas with a prevalent cribriform pattern, a morphological picture considered highly aggressive, able to determine lymphovascular and perineural invasion as well as a lower disease-free survival rate in comparison to conventional GC [2, 3].

The strength of the present paper is to achieve, for the first time, data regarding the potential prognostic behavior of HER2 status in a selected unfrequent cohort of CGC. Interestingly, the rate of HER2 amplification slightly increased in the CGC group in comparison to overall GC cohort (21.6% versus 20.6%), although, unfortunately this difference does not reach a statistically significant value. Nevertheless, the results might have been not enough robust to show a statistically significant power as a consequence of the low number of CGC cases; therefore, this latter point should be considered as a limitation of the study and requires a further validation in a larger CGC series. On the other hand, a sporadic report concerning HER2 status in CGC showed a 1+ score in 2/12 cases (16.6%) with no information about the 2+ and 3+ scores [3]; consequently, these literature data should be taken with caution due to a marked bias in selection of CGC patients.

Survival curves of all analyzed GC patients showed that patients with HER2 amplification had a shorter survival time. This worse prognosis was further confirmed in the analysis of CGC group in relation to HER2 status. Moreover, when CGC were censored according HER2 overexpression in amplified and unamplified cases, a significant prognostic HER2-positive value emerged. This relevant prognostic feature was greatly stressed, either in univariate or in multivariate analysis, in which HER2 amplification appeared as one of the main independent prognostic predictors of the cancer-related deaths together with tumor stage. However, it has been suggested that the occurrence of cribriform component, independently of its percentage, is associated with decreased overall survival and higher local recurrence, even if these data are coming from a retrospective small study group, with a not particularly prolonged follow-up period (29.7 months) [3]. Therefore, our data strongly support the peculiar behavior of HER2 status in CGC after the present check in a larger casuistry with a more extended follow-up time (34.24 months). Nevertheless, additional investigations are required, to fully elucidate the biological reasons for the strong association between HER2 overexpression and CGC variant.

However, it has been reported that diffuse GC as well as lobular breast carcinomas exhibited a reduced HER2 rate in comparison to frequent mutations of E-cadherin, with an inverse association [11, 22–24]; by contrast, in more than 50% of invasive cribriform breast carcinomas, a linear direct association between HER2 positivity and E-cadherin immunoreactivity has been already documented [25]. Moreover, a specific molecular signature for cribriform predominant carcinomas, mainly of lung origin, has been considered difficult to be found [5], while it has revealed high rates of KRAS and no EGFR mutations [26]. On this way, due to the controversy over GC classification regarding cribriform variant, supplementary studies are needed to verify a specific association of molecular signature with HER2 amplification in CGC patients.

## 5. Conclusions

Taking into practical evaluation the cribriform pattern as a paradigmatic morphological adverse prognostic factor in GC patients, also documented by its high HER2 overexpression rate, we invite all pathologists in their daily practice to specify the occurrence of cribriform neoplastic component, either in surgical or in bioptical samples, in order to correctly treat these patients with worse behavior.

## Figures and Tables

**Table 1:** Clinicopathological parameters in relation to HER2 status in 100 gastric carcinomas cases.

Parameter	Number	HER2 overexpression (%)	*p* value
Sex			*NS*
Male	48	9 (18.8)	
Female	52	12 (23.1)	
Location			*NS*
Upper third	8	1 (12.5)	
Middle third	35	9 (25.7)	
Lower third	57	11 (19.3)	
Histological type			*NS*
Cribriform	37	8 (21.6)	
No cribriform	63	13 (20.6)	
Stage			0.014
II	69	9 (13.0)	
III	28	11 (39.3)	
IV	3	1 (33.3)	
Clinical course			0.009
Alive	39	3 (7.7)	
Death from gastric cancer	61	18 (29.5)	

*NS*: not significant.

**Table 2:** Prognostic parameters examined in gastric carcinoma cases: a univariate analysis of cancer-specific mortality by Mantel-Cox log-rank test.

Variable	*X* ^2^	*df*	*p* value
All patients (*n* = 100)			
Sex	2.241	1	*NS*
Histological type	1.747	1	*NS*
Stage	41.721	1	0.000
*HER2* status	45.754	1	0.000
CGC patients (*n* = 37)			
Sex	0.219	1	*NS*
Stage	14.182	1	0.000
*HER2* status	39.973	1	0.000

*NS*: not significant; *df*: degrees of freedom.

**Table 3:** Multivariate survival analysis by Cox regression model gastric carcinoma cases.

Variable	*β*	SE	*Exp(β)*	*p* value
All patients (*n* = 100)				
Stage	1.433	0.273	4.193	0.000
*HER2* status	1.665	0.327	5.284	0.000
CGC patients (*n* = 37)				
Stage	1.603	0.546	4.970	0.003
*HER2* status	2.402	0.738	11.041	0.001

*β*: regression coefficient; SE: standard error: *Exp(β)*: ratio of risk.

## References

[1] Kadota K., Yeh Y. C., Sima C. S. (2014). The cribriform pattern identifies a subset of acinar predominant tumors with poor prognosis in patients with stage I lung adenocarcinoma: a conceptual proposal to classify cribriform predominant tumors as a distinct histologic subtype. *Modern Pathology*.

[2] Branca G., Ieni A., Barresi V., Tuccari G., Caruso R. A. (2017). An updated review of cribriform carcinomas with emphasis on histopathological diagnosis and prognostic significance. *Oncology Reviews*.

[3] Lino-Silva L. S., Salcedo Hernández R. A., Molina-Frías E. (2013). Mixed gastric carcinoma with intestinal and cribriform patterns: a distinctive pathologic appearance associated with poor prognosis in advanced stages and a potential mimicker of metastatic breast carcinoma. *International Journal of Surgical Pathology*.

[4] Kir G., Sarbay B. C., Gümüş E., Topal C. S. (2014). The association of the cribriform pattern with outcome for prostatic adenocarcinomas. *Pathology - Research and Practice*.

[5] Mackinnon Jr A. C., Luevano A., de Araujo L. C., Rao N., Le M., Suster S. (2014). Cribriform adenocarcinoma of the lung: clinicopathologic, immunohistochemical, and molecular analysis of 15 cases of a distinctive morphologic subtype of lung adenocarcinoma. *Modern Pathology*.

[6] Sarbay B. C., Kir G., Topal C. S., Gumus E. (2014). Significance of the cribriform pattern in prostatic adenocarcinomas. *Pathology - Research and Practice*.

[7] Lino-Silva L. S., Salcedo-Hernández R. A., Herrera-Gómez A. (2014). Colonic cribriform carcinoma, a morphologic pattern associated with low survival. *International Journal of Surgical Pathology*.

[8] Bosman F. T., Carneiro F., Hruban R. H., Theise N. D. (2010). *WHO Classification of Tumours of the Digestive System*.

[9] Bang Y. J., Van Cutsem E., Feyereislova A. (2010). Trastuzumab in combination with chemotherapy versus chemotherapy alone for treatment of HER2-positive advanced gastric or gastro-oesophageal junction cancer (ToGA): a phase 3, open-label, randomised controlled trial. *The Lancet*.

[10] Ieni A., Barresi V., Giuffrè G. (2013). HER2 status in advanced gastric carcinoma: a retrospective multicentric analysis from Sicily. *Oncology Letters*.

[11] Ieni A., Barresi V., Rigoli L., Caruso R. A., Tuccari G. (2015). HER2 status in premalignant, early, and advanced neoplastic lesions of the stomach. *Disease Markers*.

[12] Giuffrè G., Ieni A., Barresi V., Caruso R. A., Tuccari G. (2012). HER2 status in unusual histological variants of gastric adenocarcinomas. *Journal of Clinical Pathology*.

[13] Ieni A., Barresi V., Caltabiano R. (2014). Discordance rate of HER2 status in primary gastric carcinomas and synchronous lymph node metastases: a multicenter retrospective analysis. *International Journal of Molecular Sciences*.

[14] He C., Bian X. Y., Ni X. Z. (2013). Correlation of human epidermal growth factor receptor 2 expression with clinicopathological characteristics and prognosis in gastric cancer. *World Journal of Gastroenterology*.

[15] Janjigian Y. Y., Werner D., Pauligk C. (2012). Prognosis of metastatic gastric and gastroesophageal junction cancer by HER2 status: a European and USA international collaborative analysis. *Annals of Oncology*.

[16] Grabsch H., Sivakumar S., Gray S., Gabbert H. E., Müller W. (2010). HER2 expression in gastric cancer: rare, heterogeneous and of no prognostic value - conclusions from 924 cases of two independent series. *Cellular Oncology*.

[17] Marx A. H., Tharun L., Muth J. (2009). HER-2 amplification is highly homogenous in gastric cancer. *Human Pathology*.

[18] Ruschoff J., Hanna W., Bilous M. (2012). HER2 testing in gastric cancer: a practical approach. *Modern Pathology*.

[19] Shan L., Ying J., Lu N. (2013). HER2 expression and relevant clinicopathological features in gastric and gastroesophageal junction adenocarcinoma in a Chinese population. *Diagnostic Pathology*.

[20] Bartley A. N., Washington M. K., Colasacco C. (2017). HER2 testing and clinical decision making in gastroesophageal adenocarcinoma: guideline from the College of American Pathologists, American Society for Clinical Pathology, and the American Society of Clinical Oncology. *Journal of Clinical Oncology*.

[21] Barresi V., Giuffrè G., Caruso R. A., Tuccari G. (2013). HER2 status in rarer histologic types of gastric adenocarcinomas. *Archives of Pathology & Laboratory Medicine*.

[22] Zheng Z. H., Sun X. J., Ma M. C., Hao D. M., Liu Y. H., Sun K. L. (2003). Studies of promoter methylation status and protein expression of E-cadherin gene in associated progression stages of gastric cancer. *Yi Chuan Xue Bao*.

[23] Gravalos C., Jimeno A. (2008). HER2 in gastric cancer: a new prognostic factor and a novel therapeutic target. *Annals of Oncology*.

[24] Baniak N., Senger J. L., Ahmed S., Kanthan S. C., Kanthan R. (2016). Gastric biomarkers: a global review. *World Journal of Surgical Oncology*.

[25] Chintamani, Rekhi B., Bansal A., Bhatnagar D., Saxena S. (2010). Expression of E-cadherin in breast carcinomas and Its association with other biological markers – a prospective study. *Indian Journal of Surgical Oncology*.

[26] Warth A., Muley T., Kossakowski C. (2015). Prognostic impact and clinicopathological correlations of the cribriform pattern in pulmonary adenocarcinoma. *Journal of Thoracic Oncology*.

